# Reliability of genomic variants across different next-generation sequencing platforms and bioinformatic processing pipelines

**DOI:** 10.1186/s12864-020-07362-8

**Published:** 2021-01-19

**Authors:** Stephan Weißbach, Stanislav Sys, Charlotte Hewel, Hristo Todorov, Susann Schweiger, Jennifer Winter, Markus Pfenninger, Ali Torkamani, Doug Evans, Joachim Burger, Karin Everschor-Sitte, Helen Louise May-Simera, Susanne Gerber

**Affiliations:** 1 Institute of Human Genetics, University Medical Center of the Johannes Gutenberg-University Mainz, Mainz, Germany; 2 Institute of Developmental Biology and Neurobiology, Johannes Gutenberg-University Mainz, Mainz, Germany; 3 Leibniz Institute for Resilience Research, Mainz, Germany; 4 Department of Molecular Ecology, Senckenberg Biodiversity and Climate Research Centre, Senckenberganlage 25, 60325 Frankfurt am Main, Germany; 5 Institute for Molecular and Organismic Evolution, Johannes Gutenberg-University Mainz, Johann-Joachim-Becher-Weg 7, 55128 Mainz, Germany; 6 LOEWE Centre for Translational Biodiversity Genomics, Senckenberg Biodiversity, and Climate Research Centre, Senckenberganlage 25, 60325 Frankfurt am Main, Germany; 7 Department of Integrative Structural and Computational Biology, Scripps Research Translational Institute, California Campus, San Diego, USA; 8 Institute of Anthropology, Johannes Gutenberg-University Mainz, Mainz, Germany; 9 Institute of Physics, Johannes Gutenberg-University Mainz, Mainz, Germany; 10 Institute of Molecular Physiology, Johannes Gutenberg-University Mainz, Mainz, Germany

**Keywords:** Next-generation sequencing (NGS) technologies, Platform-biases, Healthy aging, Illumina, Wellderly, Longevity, Complete genomics, Aging, GATK

## Abstract

**Background:**

Next Generation Sequencing (NGS) is the fundament of various studies, providing insights into questions from biology and medicine. Nevertheless, integrating data from different experimental backgrounds can introduce strong biases. In order to methodically investigate the magnitude of systematic errors in single nucleotide variant calls, we performed a cross-sectional observational study on a genomic cohort of 99 subjects each sequenced via (i) Illumina HiSeq X, (ii) Illumina HiSeq, and (iii) Complete Genomics and processed with the respective bioinformatic pipeline. We also repeated variant calling for the Illumina cohorts with GATK, which allowed us to investigate the effect of the bioinformatics analysis strategy separately from the sequencing platform’s impact.

**Results:**

The number of detected variants/variant classes per individual was highly dependent on the experimental setup. We observed a statistically significant overrepresentation of variants uniquely called by a single setup, indicating potential systematic biases. Insertion/deletion polymorphisms (indels) were associated with decreased concordance compared to single nucleotide polymorphisms (SNPs). The discrepancies in indel absolute numbers were particularly prominent in introns, Alu elements, simple repeats, and regions with medium GC content. Notably, reprocessing sequencing data following the best practice recommendations of GATK considerably improved concordance between the respective setups.

**Conclusion:**

We provide empirical evidence of systematic heterogeneity in variant calls between alternative experimental and data analysis setups. Furthermore, our results demonstrate the benefit of reprocessing genomic data with harmonized pipelines when integrating data from different studies.

**Supplementary Information:**

The online version contains supplementary material available at 10.1186/s12864-020-07362-8.

## Background

From sequencing over variant calling to subsequent statistical analysis – variation can be introduced at any step of the genomics workflow. Each sequencing technology produces its own imprint of systematic biases, imposing one of the most crucial bottlenecks in genomics research. Sequencing is susceptible to high- and low-GC regions, as well as long homopolymer runs. Repetitive regions were also a main cause of uncertainty when assessing trio-samples for inconsistent mendelian errors [1]. Furthermore, Lam and colleagues demonstrated the discrepancy in sequencing accuracy between platforms based on one particular individual, revealing tens of thousands of platform-specific calls [2]. Another critical factor that can contribute to the variance between studies is the usage of heterogenous bioinformatic pipelines. To this end, several studies have been conducted. For example, O’Rawe et al. sequenced exomes and whole genomes from 15 individuals. Subsequent bioinformatics analysis with different pipelines found generally low concordance between data sets [3]. In 2015, three studies analyzed the NA12878 sample from the 1000 Genomes Project with slightly alternate setups and pipelines, yielding somewhat different recommendations [4–6]. More recently, in 2019, Kumaran et al. and Chen et al. both re-examined whole-exome sequencing data (WES) from NA12878, although the latter also compared whole-genome sequencing (WGS) [7, 8]. Hwang et al. compared both the European NA12878 and the African NA19240 samples from the 1000 Genomes Project. The authors found a pipeline consisting of BWA-MEM and subsequently the Genome Analysis ToolKit (GATK) with the Haplotype Caller to be sufficient to reliably detect variants in most regions, except for rare variants and difficult regions, as for example, simple repeats [9]. These studies provide evidence that processing pipelines and variant calling algorithms directly contribute to the heterogeneity observed when comparing the results from different sequencing experiments.

While the previous examples already offer important insights over a multitude of sequencing platforms and bioinformatics pipelines over the years, they also highlight the inconsistencies. Most of all, a vast majority of the previous studies were conducted on a single reference individual or at most only on a small number of individuals, not allowing to achieve generalizable conclusions that can be translated to large cohorts. These heterogeneities make it difficult to apply any filtering to a whole sequencing cohort. A common strategy before conducting Genome-Wide Association Studies (GWAS), for example, is to apply several sequencing cohort-specific filters to reduce variances, such as missingness, minor allele frequency (MAF), and departure from Hardy-Weinberg-Equilibrium (HWE) filters [10]. However, this strategy does not facilitate the analysis of potentially relevant rare variants with low frequencies, sensitive to technical errors, especially in somatic single nucleotide variants (SNV) and indel prediction methods [11]. In contrast, the detection of low-frequency variants in rare disease studies is achieved by deep sequencing and specialized data analyses. A crucial question remains which sequencing and analysis strategies can detect rare variants with high confidence in standard bioinformatics setups [12].

Additionally, most analyses gravitate towards using the same references, as, for example, provided by the Genome In A Bottle (GIAB) Consortium [13, 14]. This is a repository of well described, widely accepted, gold-standard variants. Such high-confidence variants are valuable and indispensable for benchmarking different sequencing technologies and bioinformatics pipelines. However, they also fail to represent the full spectrum of sequence cohort heterogeneity. Therefore, the assessment of additional non-gold standard data sets can solidify observations made on the gold standard variants and uncover potential differences.

To this end, we analyzed the variant call format (vcf) files from an extensive European reference data set from a cohort of 99 individuals associated with a healthy aging phenotype [15]. All subjects were sequenced three times via different technologies, namely (1.) Illumina HiSeq X (HSX), (2.) Illumina HiSeq (MOL) and (3.) Complete Genomics (CG) and processed with the respective mapping and variant calling pipelines. Furthermore, we reprocessed the Illumina cohorts’ sequencing data with BWA/GATK following GATK’s currently recommended best practice strategy for variant calling.

In order to assess heterogeneity between different sequencing and bioinformatic processing setups, we examined the concordance of SNPs and indels in distinct genomic areas such as introns, exons, intergenic regions, repeat elements, and genomic bins with varying GC content. Furthermore, we analyzed the distributions and reliability of variants with a MAF < 5% and HWE < 5% as detected under each experimental approach. Such SNPs and indels might be potentially disease-related variants that are often the focus of biomedical research.

## Results

**Fig. 1:**
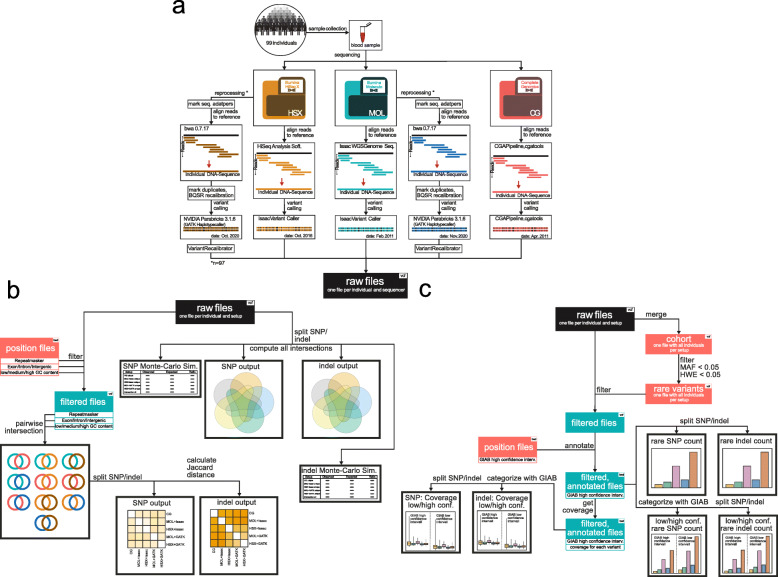
Graphical abstract of the study. **a** Schematic overview of the workflow of our analysis. 99 individuals with the wellderly phenotype were sequenced three times with Illumina HiSeq X (HSX, Apr. 2016), Illumina HiSeq (MOL, Feb. 2011), and Complete Genomics (CG, Apr. 2011). The resulting sequencing data were processed with three different bioinformatic pipelines. For CG, the in-house software cgatools (v. 1.6.0) with the Complete Genomics Analysis pipeline (v. 2.0.22) was used. At the same time, both Illumina cohorts were aligned and called with Isaac Alignment Software and Isaac Variant Caller. In our current study, we analyzed the resulting vcf-files. We also reprocessed the MOL and HSX cohorts with the GATK pipeline for germline short variant discovery (SNP + indel). **b** We evaluated the concordance of called variants between different setups by intersecting the respective vcf-files. The average number of concordant variants for different intersections was calculated and reported. Furthermore, we investigated the concordance in introns, exons, intergenic regions, repeat elements annotated with the RepeatMasker software, and bins with varying GC content. Concordance was assessed with the Jaccard distance. **c** We applied the MAF 5% and HWE 5% filters and kept the variants with MAF < 0.05 and HWE < 0.05. We then determined the distribution of these rare variants

In the current study, we re-examined the genomes of 99 individuals from the Wellderly project [15]. The wellderly phenotype describes individuals over 80 who present without any known chronic diseases and do not take any regular medication. Each individual had already been sequenced with three different next-generation sequencing platforms, namely Complete Genomics (CG), Illumina HiSeq (MOL), and Illumina HiSeq X (HSX). Variant calling had already been performed via cgatools for CG and the Isaac software for the Illumina cohorts (Fig. 1a). Therefore, the raw data in our current study were the resulting vcf files. We only considered variants, i.e., SNPs and indels with filter tag ‘PASS’ as defined by the variant calling pipeline, without equalizing the ‘PASS’ criteria between different setups. We believe that this is the most realistic approach when comparing datasets generated by different methods. Additionally, we left-aligned the variants and split multiallelic variants into consecutive blocks to equalize the variant annotation between the different sequencing cohorts.

In order to disentangle the impact of the bioinformatic processing pipeline from the sequencing technology on the concordance of variant calls, we reprocessed the Illumina sequencing data with GATK, following the current best practice recommendations for variant calling (Fig. 1a). However, due to proprietary data formats, we could not obtain and reprocess the raw data for the CG cohort. Throughout the study, we use the name of the sequencing platform (HSX, MOL, or CG) in combination with the mapping and variant calling pipeline to refer to the five distinct experimental setups we compared, namely CG, MOL + Isaac, HSX + Isaac, MOL + GATK, and HSX + GATK.

Our comparative analysis consisted of three different investigations. First, we determined the absolute number of variants in the respective experimental setups and all possible intersections. Subsequently, we analyzed the concordance of different setups within genomic regions, including exons, introns, repetitive elements, or genomic bins with varying GC content (Fig. 1b). Finally, we focused on the subset of variants filtered out after applying the MAF 5% and HWE 5% filters. We compared the distribution and reliability of such variants between the different experimental setups (Fig. 1c).

### Comparison of concordant variants between the three platforms

In the first step of the analysis, we estimated the number of variants detected in each setup. We assessed the concordance between the five experimental approaches (pipeline details are summarized in Fig. 1b).

**Fig. 2:**
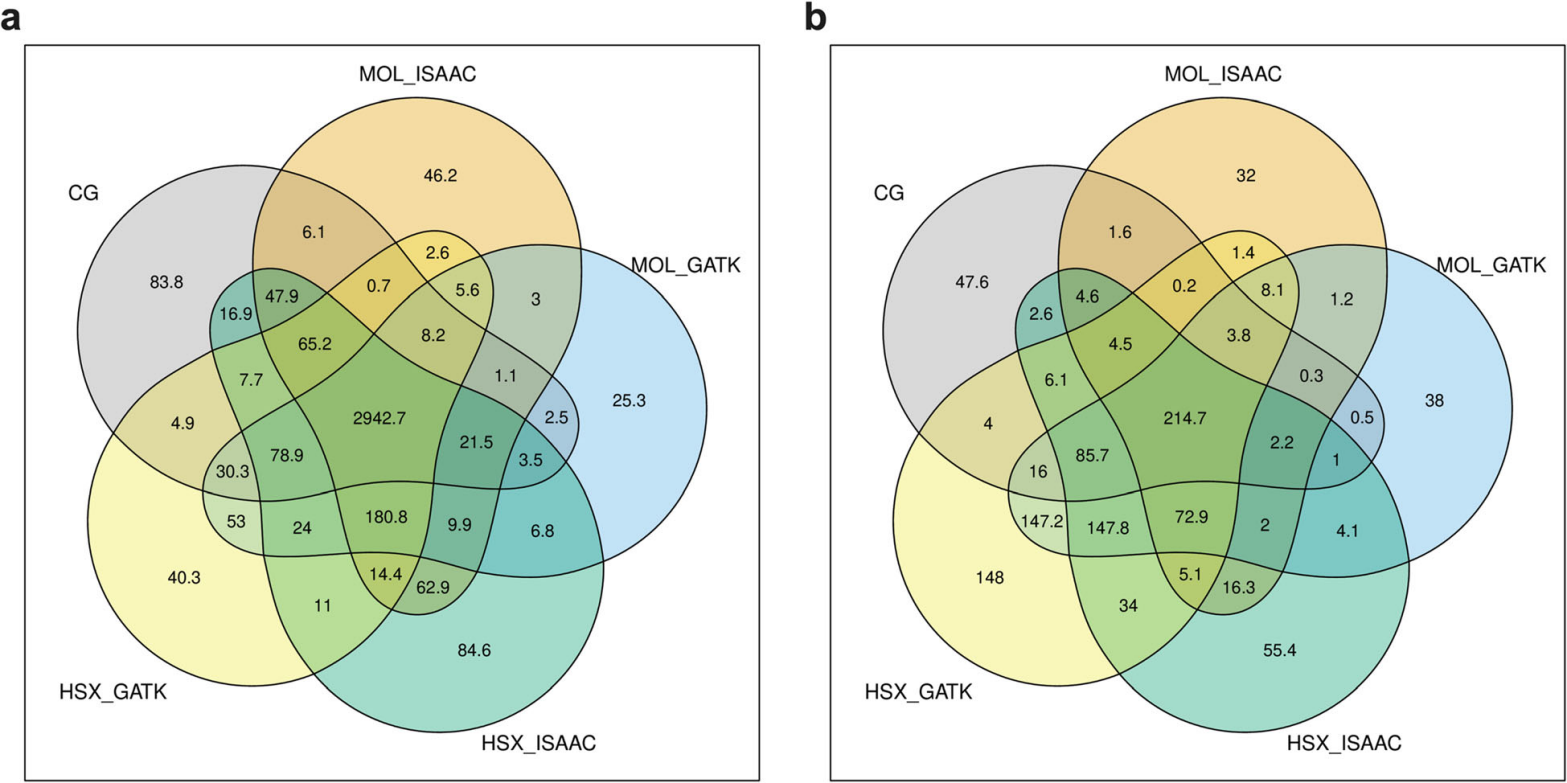
Composition of variants detected under different experimental setups. The Venn-diagrams show all possible intersections for the sets of **a)** SNPs and **b)** indels detected for each experimental setup. The quantity of variants in each subset is reported as absolute numbers divided by 1000

HSX + Isaac was associated with the highest average number of SNPs, followed by HSX + GATK, MOL + Isaac, MOL + GATK, and then CG (Fig. 2a). Altogether, an average of 2,942,659 SNPs was detected per individual by all methods, which corresponds to 82.2% of HSX + Isaac SNPs, 84.8% of HSX + GATK SNPs, 86.06% of MOL + Isaax SNPs, 86.6% of MOL + GATK SNPs, and 88.6% of CG SNPs. With respect to indels, the highest number was detected by HSX + GATK, followed by MOL + GATK, HSX + Isaac, CG, and finally MOL + Isaac (Fig. 2b). The average number of indels seen in all setups was 214,730, corresponding to 23.9% of HSX + GATK indels, 28.8% of MOL + GATK indels, 32.5% of MOL + GATK indels, 54.3% of CG indels, and 57.9% of MOL + Isaac indels.

We tested for the statistical over- and underrepresentation of observed unique variant calls for each setup and their intersection (Table 1) with a Monte Carlo simulation approach. The distributions of the observed number of SNPs and indels differed significantly form the expected numbers (chi-square test, *p* < 2.2 × 10^− 16^ for both SNPs and indels). The intersection between all setups contained 1.41 (95% confidence interval 1.409 to 1.41) times more SNPs and 5.01 (95% confidence interval 4.97 to 5.05) times more indels than expected by chance (Table 1), increasing the confidence in these calls. However, the variants unique to each platform were highly overrepresented, especially in the case of SNPs, as demonstrated by expected-versus-observed-number-of-variants ratios that were significantly higher than 1 in all cases (Table 1). This indicated the presence of experimental-setup-specific systematic biases in variant calls.

**Table 1:** Monte Carlo simulation-based comparison of the observed and expected number of variants uniquely detected by each experimental approach and in the intersection of all setups. The difference between the observed and expected distributions was significant for both SNPs and indels (chi-square test, *p* < 2.2 × 10^− 16^). Furthermore, the observed versus expected ratios of unique variants under each setup and in the intersection of all setups were all significantly higher than 1, as indicated by the 95% confidence intervals. CI: Confidence interval, CG: Complete Genomics; MOL: Illumina HiSeq; HSX: Illumina HiSeq X

Setup	SNPs			indels		
	Observed	Expected	Ratio (95% CI)	Observed	Expected	Ratio (95% CI)
**CG unique**	83,820	449	186.51 (170.71 to 205.96)	47,640	6602	7.22 (7.05 to 7.38)
**MOL + Isaac unique**	46,188	556	83.05 (76.22 to 90.75)	31,978	5994	5.33 (5.21 to 5.47)
**HSX + Isaac unique**	84,587	881	96.05 (90.08 to 102.78)	55,376	17,455	3.17 (3.13 to 3.22)
**MOL + GATK unique**	25,309	530	47.75 (43.94 to 52.18)	37,960	24,442	1.55 (1.54 to 1.57)
**HSX + GATK unique**	40,320	633	63.67 (59.03 to 68.93)	148,008	51,200	2.89 (2.87 to 2.91)
**Intersection all**	2,942,659	2,087,460	1.41 (1.409 to 1.41)	214,730	42,853	5.01 (4.97 to 5.05)

### Distribution of variants along the genome

We were interested in the regional effects of variant calling across the genome. Therefore, we compared the genome-wide distribution of variants in particular areas, such as exons, introns, and intergenic regions, as well as the concordance in variant calls between the different experimental setups. Notably, only a very small proportion of variants were located in exons. However, the absolute number of exonic SNPs and indels was very similar for all investigated setups (Additional file 1, Fig. S1a, b). The majority of SNPs were found in introns, followed by intergenic regions with comparable distributions under different conditions. In contrast, the number of intronic and intergenic indels varied substantially where the HSX + Isaac, MOL + GATK, and HSX + GATK strategies detected substantially more indels. This finding implicates indels positioned in introns and intergenic regions as a potential primary source of heterogeneity between different setups.

**Fig. 3:**
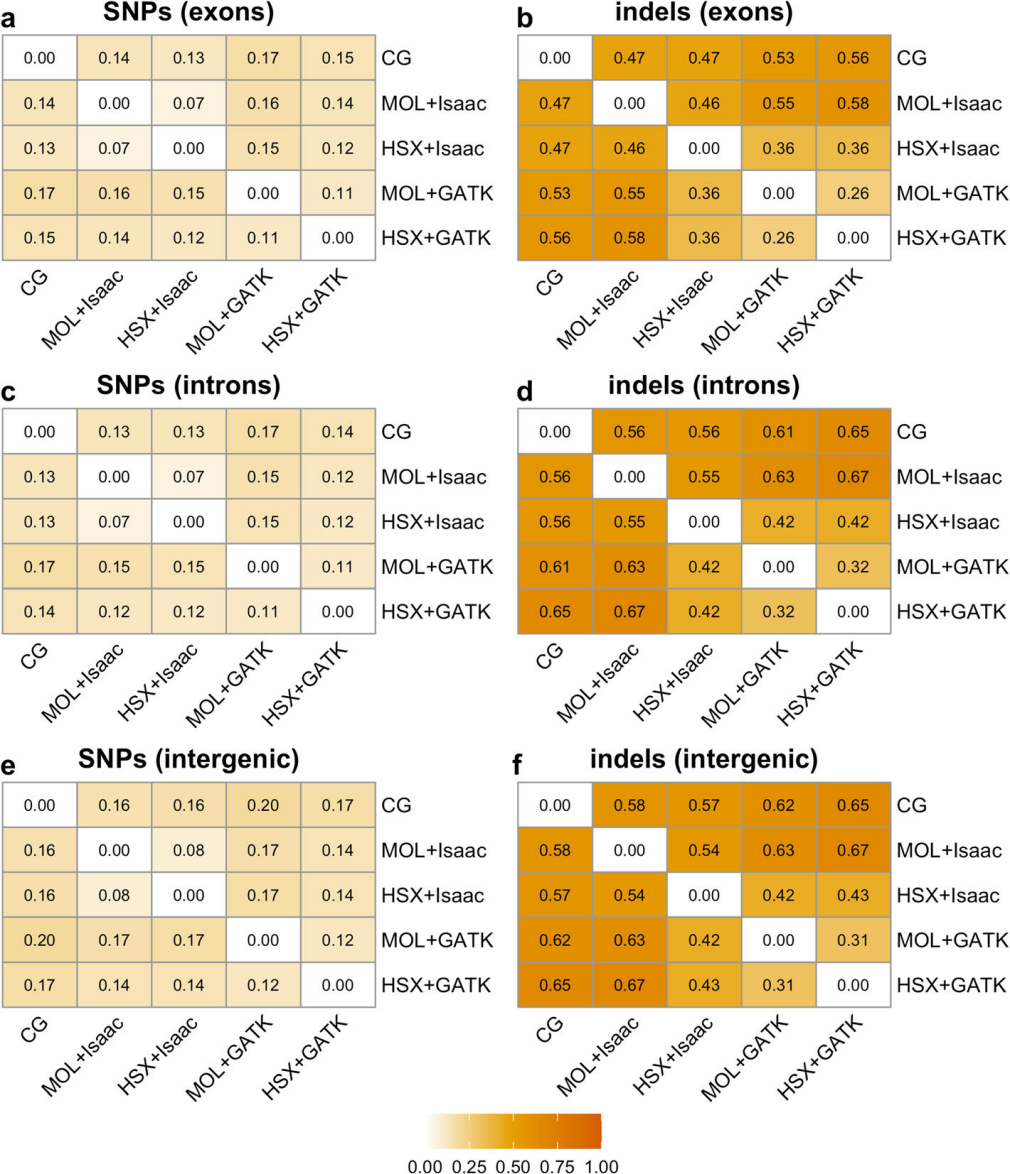
Concordance of variant calls between experimental setups in different genomic regions. Heatmaps show the pairwise concordance for SNPs (**a**, **c**, **e**) and indels (**b**, **d**, **f**) in exons, introns, and intergenic regions. Values correspond to the Jaccard distance, which ranges between 0 and 1. Higher values indicate decreased concordance between the respective experimental setups. CG: Complete Genomics; MOL: Illumina HiSeq; HSX: Illumina HiSeq X

In order to investigate the effect of genomic regions on the concordance of variant calls, we calculated the pairwise Jaccard distances between all setups. As expected, based on the distributions of the absolute numbers of variants, we observed a higher concordance for SNPs compared to indels (Fig. 3). The Jaccard distances were lowest in exons, whereas we detected the worst concordance in intergenic regions. Prominently, the best concordance for indels was achieved between MOL + GATK and HSX + GATK, which correspond to the data sets reprocessed with the currently recognized best practice mapping and variant calling strategies.

### Distribution of variants in repetitive genomic regions

Regions frequently excluded from the analysis of genomic variants are repetitive elements because they are known for accumulating sequencing errors [1]. In order to calculate the impact of such regions, we proceeded as follows (Fig. 1b): We obtained the RepeatMasker annotation for the following classes of repetitive elements, Alu elements, long interspersed nuclear elements (LINE), low complexity regions, long terminal repeats (LTR) and simple repeats. Then, we estimated the absolute number of variants detected by each setup in the respective repetitive region. Finally, we examined the pairwise concordance of variant calls between the different methods using the Jaccard distance.

We observed similar distributions for the number of SNPs in distinct repetitive regions under all experimental setups (Additional file 1, Fig. S1c). The majority of SNPs were detected in LINE, Alu, and LTR elements, whereas the number of SNPs in low complexity regions was much lower. Conversely, the absolute number of indels in the RepeatMasker annotated regions varied enormously between the setups. Notably, the highest number of indels were detected with the HSX + GATK strategy, and the difference was most prominent in Alu, LINE elements, and simple repeats (Additional file 1, Fig. S1d).

**Fig. 4:**
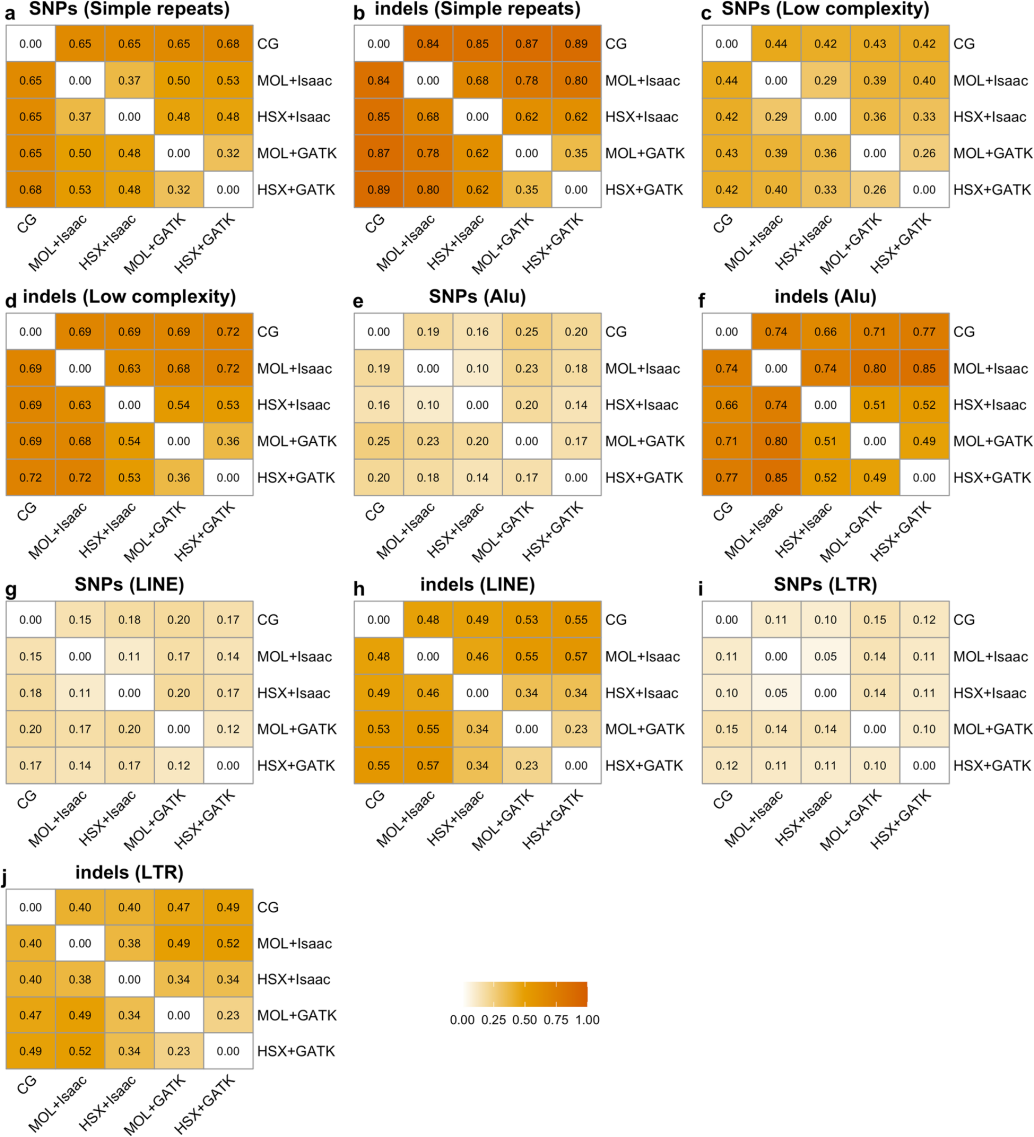
Concordance of variant calls between experimental setups in RepeatMasker regions. Heatmaps show the pairwise concordance for SNPs (**a**, **c**, **e**, **g**, **i**) and indels (**b**, **d f**, **h**, **j**) in distinct RepeatMasker regions. Values correspond to the Jaccard distance, which ranges between 0 and 1. Higher values indicate decreased concordance between the respective experimental setups. CG: Complete Genomics; MOL: Illumina HiSeq; HSX: Illumina HiSeq X; LINE: long interspersed nuclear elements; LTR: long terminal repeats

Analysis of the Jaccard distance in pairwise comparisons of experimental approaches indicated that concordance of variant calls was consistently worse for indels compared to SNPs in all types of repetitive regions (Fig. 4). This effect was most strongly pronounced in simple repeats and Alu elements with distances as high as 0.89 and 0.85, respectively (Fig. 4b and f). This finding correlates with the fact that we observed the most considerable discrepancy in the number of indels under each setup in these two types of repetitive regions (Additional File 1, Fig, S1d). Furthermore, concordance was lowest for SNPs in simple repeats compared to all other types of RepeatMasker regions (Fig. 4a). Notably, reprocessing data with the currently recognized mapping and variant calling techniques was again associated with improved concordance, particularly for indels as indicated by the lowest Jaccard distances between the MOL + GATK and HSX + GATK setups.

### Distribution of variants in genomic bins with varying GC content

GC-rich genomic regions are another potential source of sequencing errors, so they are often also excluded from the analysis of genomic variants [1]. To investigate the impact of GC content on variant detection, we calculated the GC content in genomic bins of 100 kbp. Then, we estimated the non-parametric correlation between the number of SNPs and indels in the same genomic bins with the relative proportion of GC bases.

**Fig. 5:**
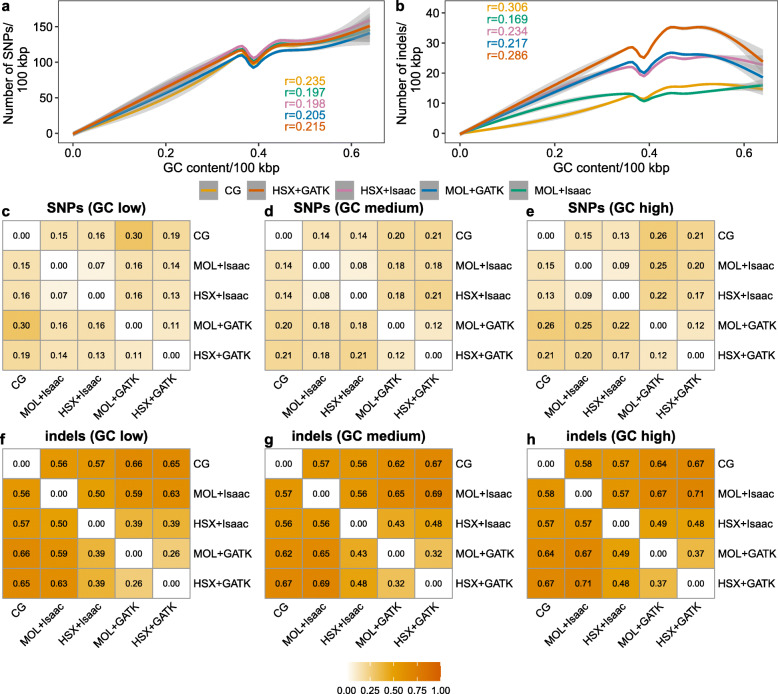
Relationship between GC content, number of variants, and concordance of variant calls between experimental setups. The correlation between GC content and the number of SNPs (**a**) and indels (**b**) based on genomic bins of 100 kbp was evaluated with the Spearman correlation coefficient r. Regression lines were fitted using generalized additive models. The correlation was highly significant for all setups (*p* < 2 × 10^− 16^). Heatmaps show the concordance between different experimental setups for SNPs (**c**, **d**, **e**) and indels (**f**, **g**, **h**) in genomic regions with low (< 37%), medium (between 37 and 47%), and high (> 47%) GC content. Values correspond to the Jaccard distance, which ranges between 0 and 1. Higher values indicate decreased concordance between the respective experimental setups. CG: Complete Genomics; MOL: Illumina HiSeq; HSX: Illumina HiSeq X

Figure 5a, b shows the trend lines for all experimental setups fitted with generalized additive models for SNPs and indels, respectively. Individual scatter plots with observed values for each approach are summarized in Additional file 1, Fig. S2a-j. We identified a weak positive correlation between the number of variants and the GC content in the 100 kbp genomic bins under all experimental conditions. This trend was more pronounced for bins with a GC content between 0% and approximately 37%. In contrast, the positive correlation between the number of variants and GC content was weaker in bins with a GC content between ~ 37% and ~ 64%. It is important to mention that the 6th percentile of the GC content distribution was already at 33%, thereby bins with a lower GC content are relatively rare (cf. Additional file 1, Fig. S2k).

In order to investigate the concordance of variant calls between experimental approaches as a function of GC content, we annotated genomic bins with a proportion of GC bases below 37% as having low, between 37 and 47% as medium, and above 47% as having high GC content (see Methods). Consequently, the majority of variants were located in regions with medium GC content (Additional file 1, Fig. S1e-f).

The Jaccard distances for SNPs and indels in the respective GC genomic regions are depicted in Fig. 5c-h. As previously observed, concordance was uniformly better for SNPs as compared with indels. The differences between regions with varying GC content were not pronounced with a slightly higher concordance in regions with a low GC content (Fig. 5c, f). Similar to the other genomic annotations (Figs. 3 and 4), the best concordance for indels was observed for the MOL-GATK vs. HSX + GATK setup.

### Distribution of rare variants and variants deviating from HWE

**Fig. 6:**
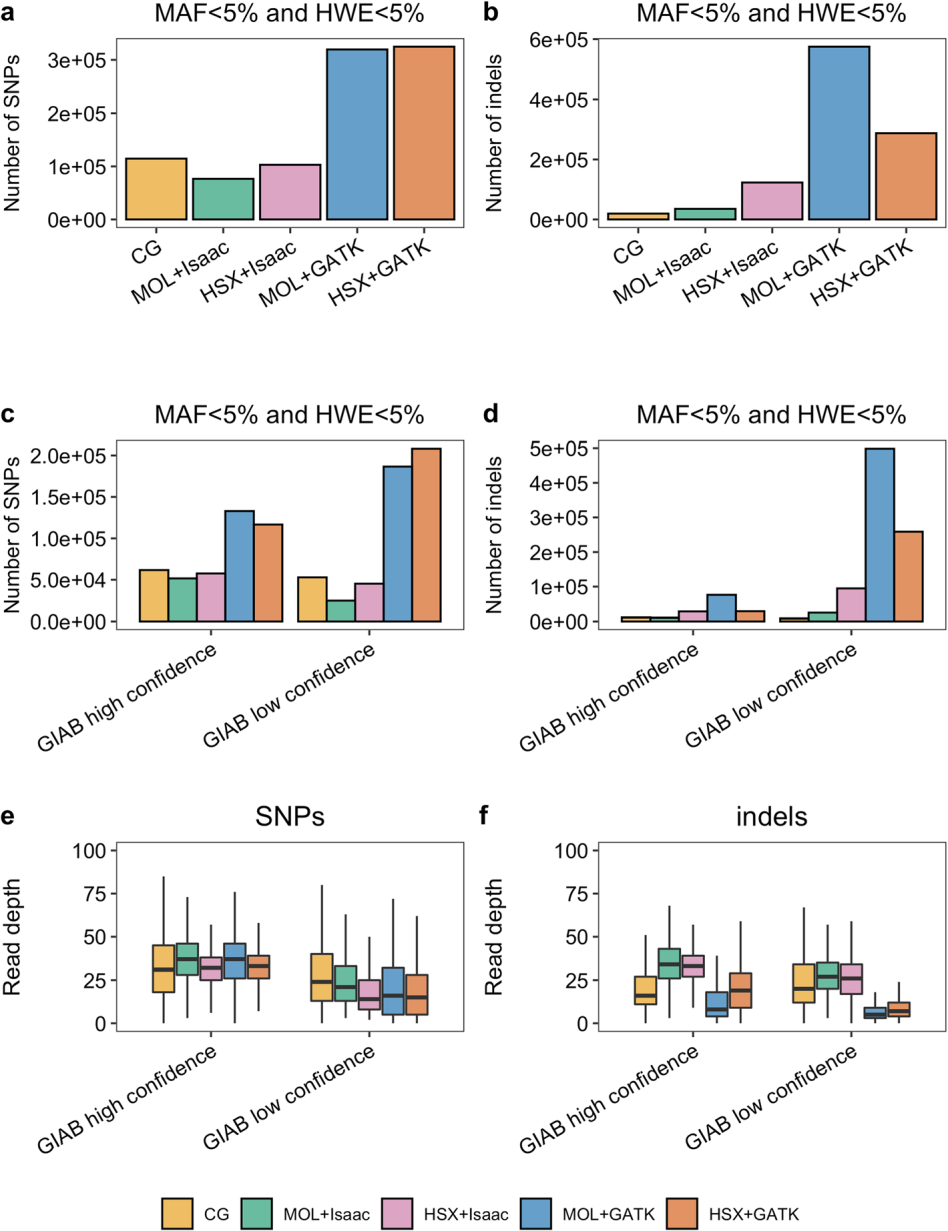
Distribution of variants failing the MAF 5% and HWE 5% filter criteria. Bar plots show the absolute number of SNPs (**a**) and indels (**b**) with MAF < 0.05 and HWE < 0.05 under each experimental condition. The distribution of such variants in high or low confidence variant call intervals according to the GIAB project is shown for SNPs (**c**) and indels (**d**). Box plots in **e**-**f** show the read depth’s distribution for variants with MAF < 0.05 and HWE < 0.05 for SNPs and indels, respectively

Applying common filters such as MAF and HWE might improve the concordance of different experimental setups. However, rare SNPs/indels and variants significantly deviating from HWE might be disease-associated which makes them particularly interesting for biomedical research. Therefore, we applied the MAF 5% and HWE 5% filters and focused on the variants which failed these two filtering criteria under each experimental setup. We observed a considerable increase in the numer of such SNPs and indels after reprocessing the sequencing data with GATK (Fig. 6a,b) compared to the remaining experimental setups. In an attempt to evaluate the realiability of these variants, we employed the GIAB annotation for high and low confidence variant call intervals. A higher number of variants in high confidence intervals could be indicative of increased sensitivity for the respective setup. The MOL + GATK and HSX + GATK setups were associated with more SNPs in high but also in low confidence intervals compared to the remaining strategies. Interestingly, the CG, MOL + Isaac and HSX + Isaac setups had more SNPs located in high compared to low confidence regions (Fig. 6b) which was also true for CG indels (Fig. 6c). In contrast, all other setups were associated with considerably more indels detected in low confidence relative to high confidence GIAB intervals (Fig. 6d). As the reliability of such variant calls might be proportional to the quantity of reads supporting them, we compared the read depth distributions of SNPs and indels with MAF < 0.05 and HWE < 0.05 in high and low confidence GIAB intervals. Median read depth for SNPs was higher in high confidence intervals under all experimental setups (Fig. 6e). In contrast, MOL + GATK and HSX + GATK exhibited reduced read depth for indels in both high and low confidence intervals compared to the remaining strategies (Fig. 6f). Therefore, it remains questionable if the increased number of variants, especially indels after reprocessing with GATK points to a higher sensitivity or potentially reduced specificity.

## Discussion

The reliability of genomic variants, especially when merging multiple cohorts, is still not thoroughly evaluated, both due to the considerable heterogeneity in laboratory protocols and variant-calling pipelines. Furthermore, numerous studies have led to conflicting estimates of the accuracy of preferred analysis pipelines for sequencing data, and challenges remain in benchmarking variant call datasets [3–6, 8, 9, 16]. In contrast to previous studies, which were only able to compare a handful of genome or exome sequences, we were given the unique opportunity to analyze a larger cohort of 99 individuals. This real-world data set allowed us to perform a practical comparison of the consistency of different sequencing and variant calling methods.

First, the average number of SNPs consistently detected throughout all experimental setups corresponded to a range of 82 to 88% of SNPs for each method. The proportion of concordant indels varied between 23 and 57% of indels from individual setups. A Monte-Carlo-simulation-based statistical test revealed that the observed number of variants called by all setups was significantly higher than expected by chance. Nevertheless, the observed number of variants unique to each method was also increased considerably relative to the expected quantity, which hints at the platform- and processing-pipeline specific biases. This finding also implies that the choice of the sequencing platform and the following bioinformatic strategy directly affect variant calling and, in turn, account for between-study heterogeneity observed in downstream analyses such as GWAS [17–19].

Upon inspection of the different subtypes of variants, we observed that all experimental setups demonstrated a greater concordance in SNPs than in indel detection. Conversely, the quantity of indels varied strongly between the platforms. Notably, HSX + GATK detected considerably more indels compared to all other setups, especially in introns and ALU elements. Furthermore, indels accounted for the majority of variants unique to HSX + GATK. The discrepancies in indel numbers were far higher than the number of random mutations an individual should have. For instance, Conrad and colleagues estimated that approximately 1000 mutations per diploid genome could be introduced due to somatic mutations, far less than the discordance we observed [20]. Indels are a class of variants that are particularly prone to amass sequencing errors. These can occur either during the PCR amplification step, the sequencing reaction, or during the alignment step because the aligner may have difficulties to place a single insertion/deletion event in a highly repetitive stretch of the genome [21]. In line with this, we observed the strongest discrepancies in indel numbers in simple repeats and Alu elements, which is characteristic for this type of mutation [22]. Prominently, reprocessing the Illumina cohorts with the GATK pipeline was associated with a considerable increase in the number of indels in these repeat regions, introns, and genomic bins with medium GC content relative to the same sequencing cohort processed with the Isaac software. This finding points to a pronounced impact of both the sequencing but also the mapping and variant calling strategy on the consistency of indel detection. These discrepancies were also reflected by a reduced concordance for indels compared to SNPs under all investigated conditions.

GC-rich regions might be another source of heterogeneity in variant calls due to mapping and coverage issues, and significant sequencing platform-specific GC biases have been previously described [23]. In our current study, we identified a weak positive correlation between higher GC content and an increased number of detected variants, as well as a slightly better concordance in bins with low GC content. Therefore, our results seem to support previous findings. In a comparative study between the Illumina and CG platforms, Lam and colleagues described lower GC content for the Illumina cohort’s concordant variants [24]. In contrast, Rieber et al. demonstrated that the CG platform is less prone to a GC bias [23]. Our correlation analysis between GC content and the number of detected variants revealed a very similar relationship for all investigated setups. Therefore, we could not ascertain if one method is more susceptible to miscalls due to GC enrichment compared to the others.

Rare variants and variants which significantly deviate from HWE might be another source of heterogeneity between different setups. Interpretation and discovery of such variants, especially disease-associated ones, remains a challenging task in rare variant studies. Unfortunately, while whole genome sequencing is superior over whole exome sequencing and panel technologies in most technical aspects, it still performs worse with respect to sequencing depth, which is an essential factor for rare variant discovery [24]. Reprocessing data with best practices and most up to date bioinformatic tools had an impact on the quantity of detected low frequency variants in our current study. However, it is not clear whether these findings indicate a higher overall sensitivity of the respective setup, when taking into account that these newly discovered variants were distributed similarly in GIAB-annotated high and low confidence intervals. Furthermore, a major concern with the GIAB dataset is the exclusion of regions which are difficult to analyze with short read sequencing, inevitably leading to biases towards easy to detect variants. Subsequently, the performance of more advanced algorithms might be hampered, since they would be penalized by the GIAB truth set, which was constructed based on older technologies [25].

Taken together, the most important finding of our study is that reprocessing sequencing data produced by different platforms with a currently accepted best practice bioinformatics pipeline significantly reduces heterogeneity, especially for indels as shown by the consistently lowest Jaccard distances between the MOL + GATK and HSX + GATK setups under all investigated conditions. This observation implies that the mapping and variant calling algorithms have a more substantial impact on the homogeneity of this more challenging class of variants than the sequencing technology. This is in line with previous reports, as significant discrepancies between competing processing strategies even when using the same sequencing method have been reported before, particularly for indels [3, 26]. For instance, Cornish and colleagues identified the GATK+UnifiedGenotyper as the most sensitive strategy to detect variants among 30 alternative pipelines, and results were comparable irrespective of the aligner used [27]. Importantly, none of the strategies achieved an average sensitivity for indels higher than 33%.

Furthermore, O’Rawe et al. also reported considerable discordance between 5 different mapping and variant calling pipelines. Nevertheless, a higher proportion of indels unique to the GATK setup could be validated using amplicon sequencing compared to a SOAP-based strategy [3]. In our study, reprocessing the Illumina cohorts with GATK was associated with a significant increase in the number of detected indels. While a certain proportion of these are probably false positives, such results could be experimentally validated. In contrast, increased false negatives might be more problematic as no validation methods for undetected variants exist. Thereby, the GATK approach seems to be the recommendable strategy for indels.

Our experimental setup is not suitable for making a definitive statement about individual variants’ reliability. Individual variants could be verified with high confidence sequencing methods, such as Sanger sequencing. This technique could be used to determine an actual error rate for the different setups and to distinguish correct from incorrect variant calls in specific regions of the genome. Sensitivity and specificity of sequencing and variant calling strategies could also be evaluated by including an artificially created synthetic reference in sequencing experiments. Such references would have to include regions with high variations or regions that are difficult to align to assess challenging base-calls. However, we would like to point out that our study’s goal was not to benchmark methods but to provide practical evidence that the sequencing and bioinformatics methods introduce systematic between-study variation.

Another potential drawback of our study is that the used sequencing platforms have a legacy status and that the bulk of new data generated today stems from different platforms. Nevertheless, genomic data obtained with older sequencing technologies are now accepted resources in genomic research, such as the 1000 Genomes Project or GIAB. Furthermore, there is still a considerable number of recently published studies, which use older sequencing data from a wide variety of sources [25, 27–31] or as test-data for novel computational approaches [32, 33] and we believe this will continue to be the case. Common incentives for re-analyzing genomic cohorts include re-mapping reads to a new reference genome version [34, 35], periodic reanalysis of disease cohorts to diagnose more patients [36] or large meta-GWAS aiming to achieve statistically significant results by increasing sample sizes. Furthermore, we demonstrated that reprocessing sequencing data with the current best practice recommendations for mapping and variant calling leads to a reduced heterogeneity between data sets stemming from different sequencing platforms. However, even this strategy is limited to cases where raw sequencing data are available. For instance, in the current study, we could not reprocess the CG cohort due to the impossibility of obtaining the sequencing files.

## Conclusions

In our study, based on a cohort of 99 subjects sequenced with three different platforms, we demonstrated a considerable discordance between the sequencing technologies and bioinformatics processing pipelines. In contrast to previous studies, which have focused extensively on individuals or smaller groups, our approach using a larger cohort of 99 individuals provides a direct insight into the challenges that arise when integrating data from different sources. While variants that are uniquely detected by a single setup might point to increased sensitivity, they might also be the results of systematic errors. In agreement with previous reports, our study also highlighted the complexity of correctly calling indels, especially in tandem repeats and low complexity genomic regions. Our study suggests that while both experimental factors such as the sequencing technology as well as the choice of data analysis method considerably contribute to heterogeneous results, the impact of the mapping and variant calling strategy might be more pronounced, especially for indels. The ever-growing amount of available whole-genome sequencing data underlies the need for reliable sequencing platforms and respective bioinformatic processing pipelines. In 2001, Ioannidis and colleagues suggested that genetic studies’ meta-analyses would greatly benefit from including individual data instead of analyzing summary statistics [17]. At the time, this seemed unrealistic due to the vast collaborative data-sharing effort necessary to achieve such a goal. Currently, however, it is common practice to make raw data publicly available. While differences in the choice of sequencing platform cannot be abolished once the data have been generated, it seems prudent to reprocess raw data in a unified manner prior to conducting a meta-analysis or generally integrating data from different sources in genomic investigations. This approach would ensure more reliable and reproducible results by removing biases originating from discrepancies in the bioinformatics processing pipelines.

## Methods

### Genomes investigated

A cohort of 99 subjects with the so-called “wellderly phenotype” was investigated. The wellderly phenotype refers to individuals older than 80 years who do not have any known chronic diseases and do not receive regular medication. The subjects in our current study were sampled from a larger cohort described in Erikson et al., 2013 [15]. All individuals were sequenced three times with different sequencing technologies: (i) Complete Genomics, (ii) Illumina HiSeq X, and (iii) Illumina HiSeq with TruSeq Synthetic Long-Read DNA Library Prep Kit for long reads (Fig. 1a). All multiple nucleotide polymorphisms (MNP) were decomposed into consecutive SNP since they were not encoded as such in the vcf files of the Illumina HiSeq data set. An MNP is a variant that extends over several base pairs and has sequential bases that differ from the reference genome. We used VT decompose to split each MNP into a sequence of SNP.

Furthermore, the MOL and HSX cohorts were reprocessed bioinformatically. Therefore, the existing alignment was deleted, and the mapping and variant calling was repeated according to GATK best practice using bwa, GATK, and NVIDIA parabricks. Since two files were corrupted, they were excluded from the analysis and 97 files were used.

### Opensource tools used for analysis

**BCFtools 1.11–16** (http://samtools.github.io/bcftools/bcftools.html) was a standard analysis tool in this study. We used BCFtools for filtering (QUAL, MAF, HWE and region-based filters), querying (i.e. creation of a bed-file) and intersecting. BCFtools stats was used to retrieve information about the number of variants per sample. (SNP/indel). In addition, we worked with the plugin fill-tags to update variant tags in vcf-files (i.e., MAF, HWE). In order to join all individual vcf-files to a single cohort-level file, we used BCFtools merge. Since vcf is a reduced file format, which only includes the differences to the reference genome of a given genetic sequence, we assumed, that a missing variant corresponds to a reference genome type at this position.

### Bwa 0.7.17

The mapping of the unaligned files was done with bwa mem according to the GATK best practice. We used the flag “–M” to mark shorter, split hits as secondary and thus ensure Picard compatibility, otherwise default settings were applied.

**GATK 4.1.8.1, Picard 2.22.8, HTSJDK 2.23.0** (https://gatk.broadinstitute.org/hc/en-us).

GATK was used for the processing of the sequencing files for MOL and HSX. We used GATK to remove the existing alignment, trim adapter sequences, mark duplicates, calculate and apply BQSR and VQSR. The file processing was performed according to GATK best practice for germline short variant discovery of SNP and indel (https://gatk.broadinstitute.org/hc/en-us/articles/360035535932-Germline-short-variant-discovery-SNPs-Indels-, accessed on 2nd Nov 2020). We used the recommended files from the GATK resource bundle (https://gatk.broadinstitute.org/hc/en-us/articles/360035890811-Resource-bundle, accessed on 15th Sep 2020). To make the former vcf-files (CG + cgatools, MOL + ISAAC, HSX + ISAAC) comparable to the newly processed files (MOL + GATK, HSX + GATK), a lift-over from hg19 to hg38 was performed.

### Nvidia Clara Parabricks 3.1.6

**(****https://www.nvidia.com/en-us/docs/parabricks/quickstart-guide/software-overview/****)**

For variant calling, the NVIDIA Clara Parabricks HaplotypeCaller was used, a GPU accelerated version of the GATK HaplotypeCaller.

**Tabix 1.7.2** (http://www.htslib.org/doc/tabix.html) was used to create index files for gziped vcf-files and is needed by BCFtools for processing.

**Bedtools 2.27.1** (https://bedtools.readthedocs.io/en/latest/) was used to intersect a bed with another bed-file (Bedtools intersect). Bedtools nuc was used to calculate the GC content of 100kbp bins for the whole genome.

With **Vt 0.57721** decompose (https://github.com/atks/vt), we split MNP into consecutive SNP. This procedure was necessary because the vcf files of MOL did not include MNP.

We used **R 3.6.1** (https://www.r-project.org/) for basic calculations, data manipulation, and as a framework for plotting using ggplot2 3.2.1 (https://ggplot2.tidyverse.org/) or ggpubr.

### Tracks

We aimed to identify regions that differ more between the sequencing technologies and post-sequencing algorithms. Several annotation tracks that can be downloaded and applied easily were used for this purpose. If not already available, we converted the track into the bed-file format and processed the files with it.

Repeatmasker: Alu, LINE, low complexity, LTR, simple repeats.

(http://repeatmasker.org/genomes/hg38/RepeatMasker-rm405-db20140131/hg38.fa.out.gz, accessed on 5th Nov 2020)

Annotations for exonic, intronic, and intergenic regions were retrieved from the UCSC Browser (https://www.genome.ucsc.edu/cgi-bin/hgTables, accessed on 5th Nov 2020). Additionally, we created bed-files of the following features:

- Minor allele frequency < 0.05
- Hardy-Weinberg-Equilibrium < 0.05

#### Minor allele frequency

The Minor Allele Frequency (MAF) is a commonly used filter for downstream analysis of genetic data. The individual data of all samples were merged into a cohort file. Since vcf-format is sparse and only contains positions that differ from the reference genome, we set ‘missing positions’ as reference. The resulting file with the variants was used to filter each individual vcf-file of the respective set-up.

#### Hardy-Weinberg-equilibrium

The Hardy-Weinberg-Equilibrium (HWE) is a theoretical measure for the derivation of variants from the expected Mendelian heritage. Here, we used an HWE-threshold of *p* < 0.05. Our approach was identical, as described in the section “Minor Allele Frequency”.

#### Genome in a bottle (GIAB)

The GIAB project’s priority is the characterization of human genome material for analytical validation, optimization, and technology development [13, 14]. It is frequently used as reference material to benchmark bioinformatics tools as variant calling algorithms.

While providing high confidence calls for different human samples as NA12878, it also provides an annotation track for high confidence intervals for variant calls, which was used to characterize variants that were filtered with a MAF < 0.05 and HWE < 0.05 filter to investigate their plausibility in the different bioinformatic setups. By comparing the number of variants found in high confidence regions, one can estimate the sensitivity of distinct setups. The genomic regions complementary to the GIAB high confidence intervals were annotated as low confidence in the current study.

Annotation track for high confidence intervals:

ftp://ftp-trace.ncbi.nlm.nih.gov/giab/ftp/release/NA12878_HG001/latest/GRCh38/, accessed on 25th^.^ Nov 2020.

### GC content annotation

GC content was calculated as the proportion of GC bases in genomic bins with a length of 100 kbp using bedtools nuc. This resulted in a distribution of genomic GC content with a median of 39.4% (Additional File 1, Fig. S2k). Genomic bins were annotated as having low GC content (less than 37%), medium GC content (between 37 and 47%), and high GC content (more than 47%). These cut-off values were based on the quantiles of the GC content distribution. Namely, 37% GC content corresponds to the 30th percentile of the distribution, meaning that approximately 1/3 of the bins have a lower GC content. The next cut-off value was chosen as a middle point between 37% and the maximal value of 64% GC content. The symmetrical middle point between 37 and 64% is approximately 50%. However, this value corresponds to the 95th percentile of the observed GC content distribution. In order to balance the proportion of bins with a high GC content, we, therefore, chose the lower cut-off value of 47%, which corresponds approximately to the 90th percentile of the GC content distribution.

### Statistical analysis

The pairwise concordance between experimental setups was investigated with the Jaccard distance, which is calculated by dividing the intersection of two sets by the union of the sets and subtracting the resulting value from 1:

$$ \mathrm{Jaccard}\ \mathrm{distance}\left(\mathrm{A},\mathrm{B}\right)=1-\frac{\left(\mathrm{A}\cap \mathrm{B}\right)}{\left(\mathrm{A}\cup \mathrm{B}\right)} $$

Values of the Jaccard distance vary between 0 and 1, and lower values correspond to a higher concordance between two experimental setups.

The observed and expected number of unique variants for the five experimental setups and their intersection (Venn-diagrams in Fig. 2) was statistically compared using a Monte Carlo simulation approach. Assuming the total average number of SNPs (3,892,351) and indels (1,108,674) observed as the “true” base population from which variants were called by each experimental approach, we could infer the statistical over- or under-representation of variants uniquely called by each setup and the intersection of all setups. We computed the null-distribution for the expected number of variants in each set by randomly drawing the observed number of variants for each approach and determining the respective number of unique variants. This step was repeated 1000 times, and the mean values over all runs were taken as estimates for the null distribution of the number of unique variants. The observed number of unique variants was then compared against the expected null distribution with a chi-squared test. Furthermore, we calculated the ratio of the observed versus expected number of variants under each condition. These ratios were considered to be significantly higher than 1 if the lower bound of the 95% confidence interval did not cross 1. The confidence intervals were obtained by calculating the 2.5% and the 97.5% quantiles of the empirical distributions of the expected versus observed ratios from the 1000 simulation steps.

The correlation between the number of variants and GC content in genomic bins of 100 kbp was evaluated with the non-parametric Spearman correlation coefficient. Regression lines were fitted using generalized additive models as implemented in the stat_smooth() R function.

## Supplementary Information

**Additional file 1: Supplementary Fig. S1**: Distribution of variants along the genome. **Supplementary Fig. S2**: Correlation between GC content and number of variants detected.

## Data Availability

The raw-data that support the findings of this study are not publicly available since they contain highly sensitive personal information allowing to identify the individual subjects. Requests for obtaining the raw data should be directed to Ali Torkamani at atorkama@scripps.edu in combination with an IRB Approval. Annotation tracks for RepeatMasker region were obtained from: http://repeatmasker.org/genomes/hg38/RepeatMasker-rm405-db20140131/hg38.fa.out.gz, accessed on 5th Nov 2020. Annotation tracks for exons, intros and intergenic regions were downloaded from the UCSC Browser (https://www.genome.ucsc.edu/cgi-bin/hgTables, accessed on 5th Nov 2020). Annotations for GIAB high conficence variant call intervals were retrieved from ftp://ftp-trace.ncbi.nlm.nih.gov/giab/ftp/release/NA12878_HG001/latest/GRCh38/, accessed on 25th^.^ Nov 2020. All accession numbers or direct web links to annotation tracks for exons, introns and intergenic regions obtained from UCSC and used in this study are available for download at https://seafile.rlp.net/d/7bd50927a9a8451dbdf8/

## Abbreviations

CG: Complete Genomics
GIAB: Genome in a bottle
GWAS: Genome-wide Association Study
HSX: Illumina HiSeq X
HWE: Hardy-Weinberg-Equilibrium
indel: Insertion/Deletion Polymorphism
LINE: Long Interspersed Nuclear Elements
LTR: Long Terminal Repeats
MAF: Minor Allele Frequency
MOL: Illumina HiSeq
NGS: Next Generation Sequencing
SNP: Single Nucleotide Polymorphism
VCF: Variant Call Format
WGS: Whole-Genome Sequencing
WES: Whole-Exome Sequencing
