# Supplementary material for: Reliability of genomic variants across different next-generation sequencing platforms and bioinformatic processing pipelines
Source: BMC Genomics. 2021 Jan 19;22:62. doi: 10.1186/s12864-020-07362-8 (PMC7814447; doi:10.1186/s12864-020-07362-8)
Supplement: Supplementary file 1 — Additional file 1: Supplementary Fig. S1: Distribution of variants along the genome. Supplementary Fig. S2: Correlation between GC content and number of variants detected. [file 12864_2020_7362_MOESM1_ESM.docx]

**Reliability of genomic variants across different next-generation sequencing platforms and bioinformatic processing pipelines**

Stephan Weißbach, Stanislav Sys, Charlotte Hewel, Hristo Todorov, Susann Schweiger, Jennifer Winter, Markus Pfenninger, Ali Torkamani, Doug Evans, Joachim Burger, Karin Everschor-Sitte, Helen May-Simera and Susanne Gerber


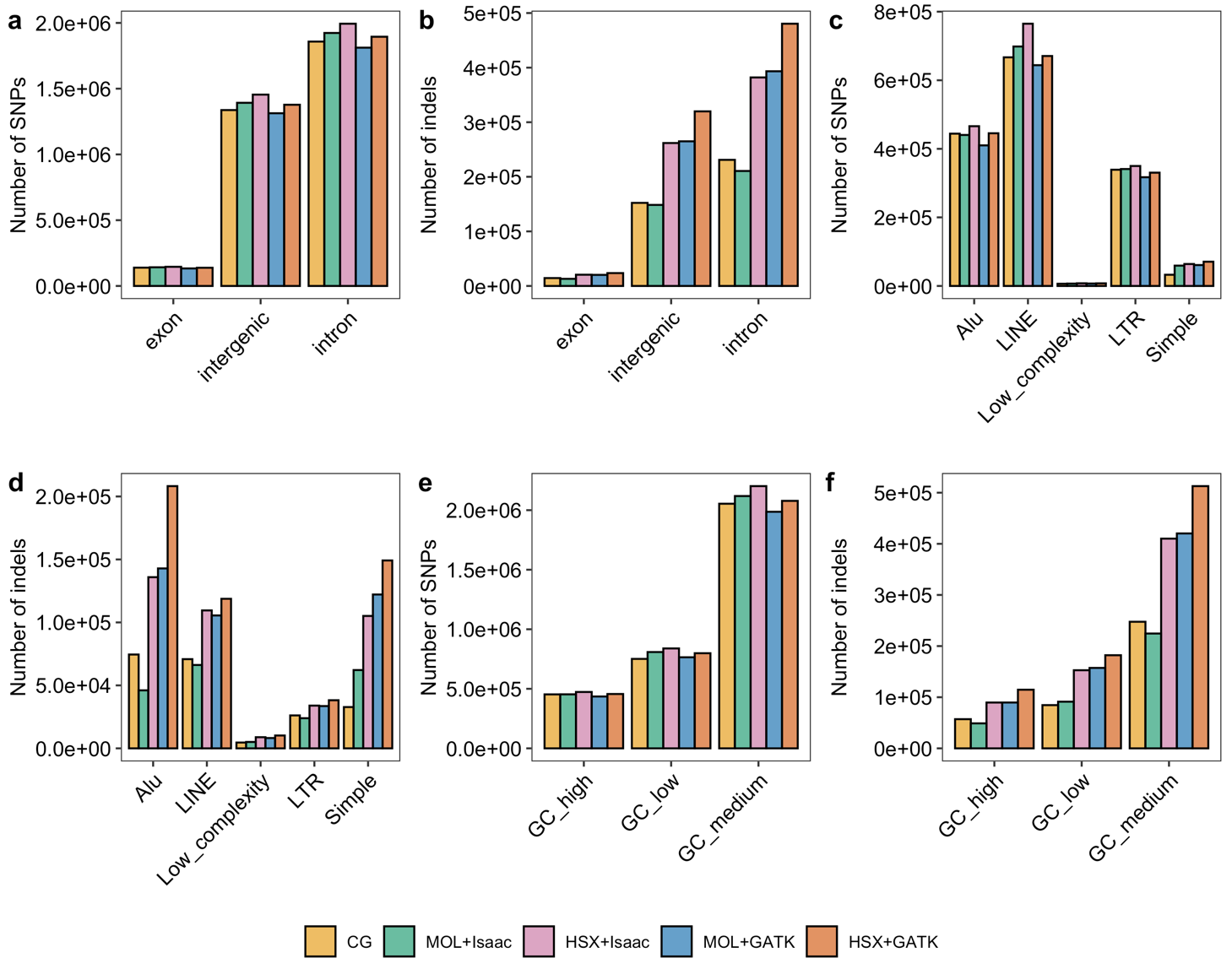


**Fig. S1: Distribution of variants along the genome.** Bar graphs show the average number of variants for each setup in different genomic regions. **a-b**) Number of SNPs and indels, respectively, in exons, introns and intergenic regions. c-d: Number of SNPs and indels, respectively, in RepeatMasker regions. **e-f)** Number of SNPs and indels, respectively, in genomic regions with low (<37% per 100 kbp), medium (between 37% and 47% per 100 kpb) and high GC (>47% per 100 kpb) GC content. CG: Complete Genomics; MOL: Illumina HiSeq; HSX: Illumina HiSeq X; LINE: long interspersed nuclear elements; LTR: long terminal repeats.


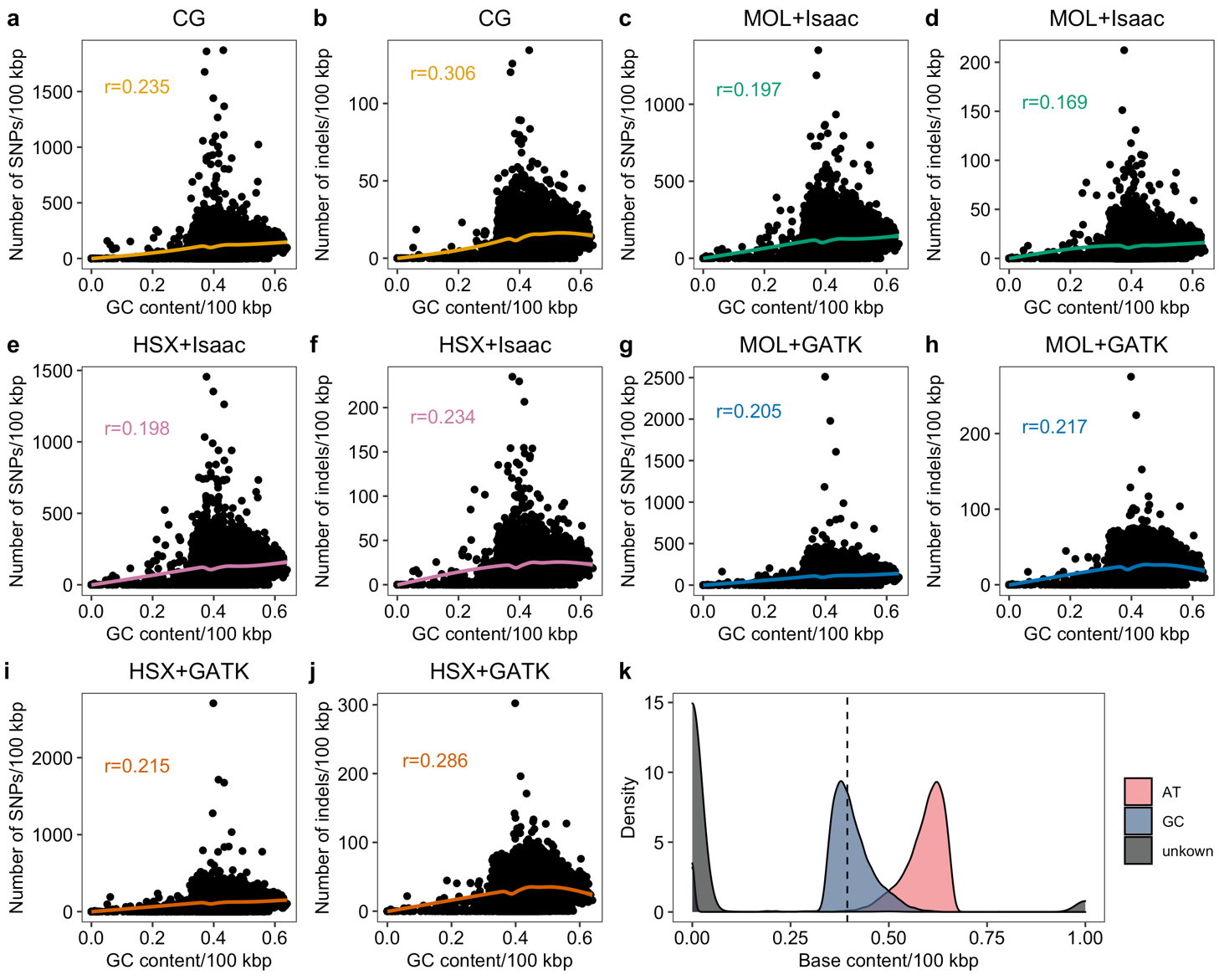


**Fig. S2: Correlation between GC content and number of variants detected.** **a-j** show the relationship between GC content and number of SNPs or indels in genomic bins of 100 kbp for each experimental setup. Regression lines were fitted using generalized additive models. The correlation coefficient r was highly significant for each experimental setup (p<2.2x10^-16^). Correlation was calculated using the non-parametric Spearman coefficient r. **k)** Distribution of the base content (AT, GC or unknown) calculated based on genomic bins of 100 kbp. The median GC content (0.394) is indicated by a vertical dashed line. CG: Complete Genomics; MOL: Illumina HiSeq; HSX: Illumina HiSeq
